# Spatially resolved longitudinal changes in tibial subchondral trabecular bone texture for exploratory prediction of incident knee osteoarthritis

**DOI:** 10.3389/fphys.2026.1904598

**Published:** 2026-09-21

**Authors:** Ahmad Almhdie-Imjabbar, Hechmi Toumi, Eric Lespessailles

**Affiliations:** 1 Plateforme Recherche Innovation Médicale Mutualisée d'Orléans (PRIMMO), University Hospital Center of Orleans, Orleans, France; 2 Department of Rheumatology, University Hospital Center, Orleans, France; 3 Faculty of Sciences, Technology, University of Orleans, Orleans, France; 4 Laboratoire des Adaptations Métaboliques à l’Exercice en Conditions Physiologiques et Pathologiques (AME2P), Université Clermont Auvergne (UCA), Aubière, France

**Keywords:** conventional radiograph, knee osteoarthritis, prediction, relative importance analysis, trabecular bone texture

## Abstract

Although prediction of radiographic knee osteoarthritis (rKOA) using baseline clinical and radiological data has been widely studied, the impact of temporal changes in imaging and clinical biomarkers remains less investigated. This study examined whether longitudinal changes in spatially resolved trabecular bone texture (TBT) improve prediction of incident rKOA. Participants with longitudinal data were selected from the Osteoarthritis Initiative. Incident rKOA was defined as Kellgren–Lawrence grade (KL) 0 at baseline and KL ≥ 2 at 48 months. One knee per participant was included (n=481). Fractal-based TBT descriptors were extracted from 16 automatically defined regions of interest across the tibial subchondral bone. Five logistic-regression models combined baseline clinical and radiological covariates, baseline TBT, and 24-month longitudinal changes (ΔTBT). Bidirectional stepwise selection based on the Akaike Information Criterion was applied once to TBT descriptors, whereas clinical and radiological covariates were incorporated *a priori*. The resulting fixed model specifications were evaluated using stratified 10-fold cross-validation repeated 300 times. Performance was assessed using area under the ROC curve (AUC), F1 score, balanced accuracy, positive and negative predictive values, and DeLong comparisons with the reference model. Adding baseline TBT to the clinical and radiographic reference model increased AUC, although not significantly (0.64 versus 0.53; p=0.055). Models incorporating ΔTBT yielded the highest apparent discrimination (AUC 0.76, 95% CI 0.70–0.82; p<0.001 versus the reference model), but threshold-dependent performance remained modest, with maximum balanced accuracy of 0.56, PPV of 0.22, and F1 score of 0.21. Selected baseline and longitudinal TBT descriptors were distributed across medial and lateral compartments, including regions near the central axis and subchondral cartilage. In this exploratory analysis, models incorporating spatially resolved 24-month TBT changes showed higher apparent ranking discrimination than the clinical and radiographic reference model. However, classification at the 0.5 threshold remained limited, and AUC improvement may be optimistic because feature selection preceded cross-validation. These findings support further investigation of ΔTBT as a candidate imaging marker but do not establish clinical utility. Leakage-free internal and external validation across independent cohorts and imaging systems are required.

## 1 Introduction

Osteoarthritis (OA) affects more than 500 million people worldwide. Moreover, due to an aging global population and the increasing burden of high Body Mass Index (BMI), projections estimate by 2050 more than one billion people will be affected (Force et al., 2025; Fuggle et al., 2025). The management of knee OA (KOA), which is the most common form of systemic OA (Whittaker et al., 2021), often leads to arthroplasty, due to the lack of disease modifying treatment. Although arthroplasty is a well-established procedure to improve quality of life, it is recognized that one patient in four suffers from persistent pain and disability after the surgery (Hunter and Bierma-Zeinstra, 2019). Currently, OA therapies are used as a one-size-fits-all approach, even though growing evidence indicates that more targeted therapies are needed and should be adapted to the specific phenotype and endotype of each patient (Tang et al., 2025). Consequently, it critically importance to intercept the disease at an early stage by identifying individuals the most at risk of developing this condition (Tang et al., 2025). Widespread KOA screening in routine clinical settings can be obtained through conventional radiography, a cost-effective alternative to magnetic resonance imaging. Trabecular bone texture (TBT) has been identified as a promising biomarker for predicting several clinically relevant KOA outcomes, including incidence, progression and the risk of total knee arthroplasty (Janvier et al., 2017a; Kraus et al., 2018; Almhdie-Imjabbar et al., 2022b; Nasser et al., 2023). Moreover, TBT-based prediction models have demonstrated strong robustness, performing consistently, both in training and validation, across two large independent cohorts (Almhdie-Imjabbar et al., 2022a). Incorporating longitudinal radiomic parameters, alongside baseline features, has been shown to further enhance predictive performance for KOA progression relative to using baseline data alone (Almhdie-Imjabbar et al., 2023b).

Based on these findings, this study aimed to evaluate whether longitudinal changes in TBT could serve as relevant descriptors to enhance the predictive performance for radiographic knee osteoarthritis (rKOA) incidence in the OAI cohort, as compared with previously established prediction models (Janvier et al., 2017b). A secondary aim was to characterize the anatomical distribution and relative importance of both baseline and longitudinal TBT features across the tibial subchondral plateau contributing to overall model performance.

The findings of this study suggest that specific regions of the subchondral proximal tibia may be particularly relevant for predicting the incidence of knee osteoarthritis, thereby providing additional insight into the pathogenic mechanisms of the disease. Whether these regional TBT changes are associated with region-specific patterns of cartilage loss within the joint merits further investigation.

## 2 Materials and methods

### 2.1 Patients

Fully anonymized data were obtained from the Osteoarthritis Initiative (OAI) public repository, a longitudinal cohort designed to identify risk factors and imaging biomarkers of the incidence and/or progression of KOA. In this cohort, bilateral posteroanterior fixed-flexion knee radiography was used to study both knees of 4796 participants during 8 years. The OAI study was carried up in accordance with all relevant guidelines and regulations. Written informed consent was obtained from all participants by the OAI investigators at the time of cohort enrollment. Details about the acquisition and grading protocols are available online at (https://nda.nih.gov/oai/study-details). Access to the raw de-identified data used in this study was approved by the OAI permission group of the National Institute of Mental Health Data Archive.

### 2.2 Data selection

Radiographic (KL and medial joint space narrowing [JSNM]) and clinical data were harmonized across baseline, 24- and 48-month visits to construct a dataset suitable for modeling incident rKOA. clinical covariates (CLIN: age, sex, body mass index [BMI], and WOMAC pain score) and radiographic descriptors.

#### 2.2.1 Radiographic eligibility

All included knees were required to have readable postero-anterior fixed-flexion radiographs at baseline, 24-month, and 48-month visits, free of artifacts, metallic implants, or incomplete tibial coverage.

To ensure that only joints at risk of developing new radiographic osteoarthritis were included, we restricted the cohort to knees with KL grade = 0 at baseline, corresponding to radiographically normal joints. This restriction excludes prevalent OA cases (KL ≥ 1) and minimizes potential misclassification of early structural changes.

Such baseline restriction is widely adopted in longitudinal incidence studies using the OAI cohort (Felson et al., 2011).

The analytical dataset included images obtained using both computed radiography and digitized conventional radiography. OAI serial knee radiographs were acquired using a standardized fixed-flexion protocol with a SynaFlexer positioning frame and centralized quality control. Participants were followed at the same clinical center, and acquisition protocol, radiographic modality, and image resolution were maintained across longitudinal visits. The analytical dataset included both computed radiography (CR) and digitized conventional radiography (RG), but acquisition modality and resolution remained consistent longitudinally within each participant. Of the included radiographs, 68% were obtained using computed radiography and 32% using digitized conventional radiography.

#### 2.2.2 Individual variability

To ensure adequate covariate availability, we further retained only participants with complete clinical covariates (CLIN), including demographic [age, sex, body mass index (BMI)], and the Western Ontario and McMaster Universities Arthritis (WOMAC) pain score data at baseline and the 24-month follow-up.

#### 2.2.3 Index-knee selection

To avoid within-participant correlation, one index knee per participant was retained for analysis. When both knees satisfied the baseline eligibility criteria, the index knee was selected using the following hierarchical rule.

- 48-month KL progression: If only one of the two eligible knees progressed to KL grade ≥2 at 48 months, the progressing knee was selected. If both or neither knee progressed, the second criterion was applied.
- Baseline pain: If both knees met the first criterion, the knee with the higher baseline WOMAC pain score (range is 0–20; a higher number represents worse symptoms) was selected as the index knee (Eckstein et al., 2011; Collins et al., 2014).
- Laterality: If both knees met the second criterion, the right knee was selected to ensure consistent decision logic across participants (Eckstein et al., 2011; Almhdie-Imjabbar et al., 2023b; van Helvoort et al., 2023).

This rule ensured that only one knee was included per participant but preferentially selected the incident knee when only one of two eligible knees developed rKOA. The potential selection bias introduced by this outcome-dependent criterion is acknowledged as a study limitation.

#### 2.2.4 Definition of OA incidence

Among these at-risk knees (KL = 0), those with KL ≥ 2 at 48-month follow-up were defined as incident rKOA, while the remaining knees served as non-incident comparators. Because 48-month progression status contributed to index-knee selection when both knees were eligible, the resulting case proportion should not be interpreted as an unbiased estimate of population incidence.

For an inherent fairness in the comparison of prediction models based on 24-month changes in clinical and radiological values, incident knees within 24 months (presenting KL > 1 at 24-month follow-up) were excluded. **Figure 1** illustrates the methodological framework used for case–control selection in this study.

**Figure 1:**
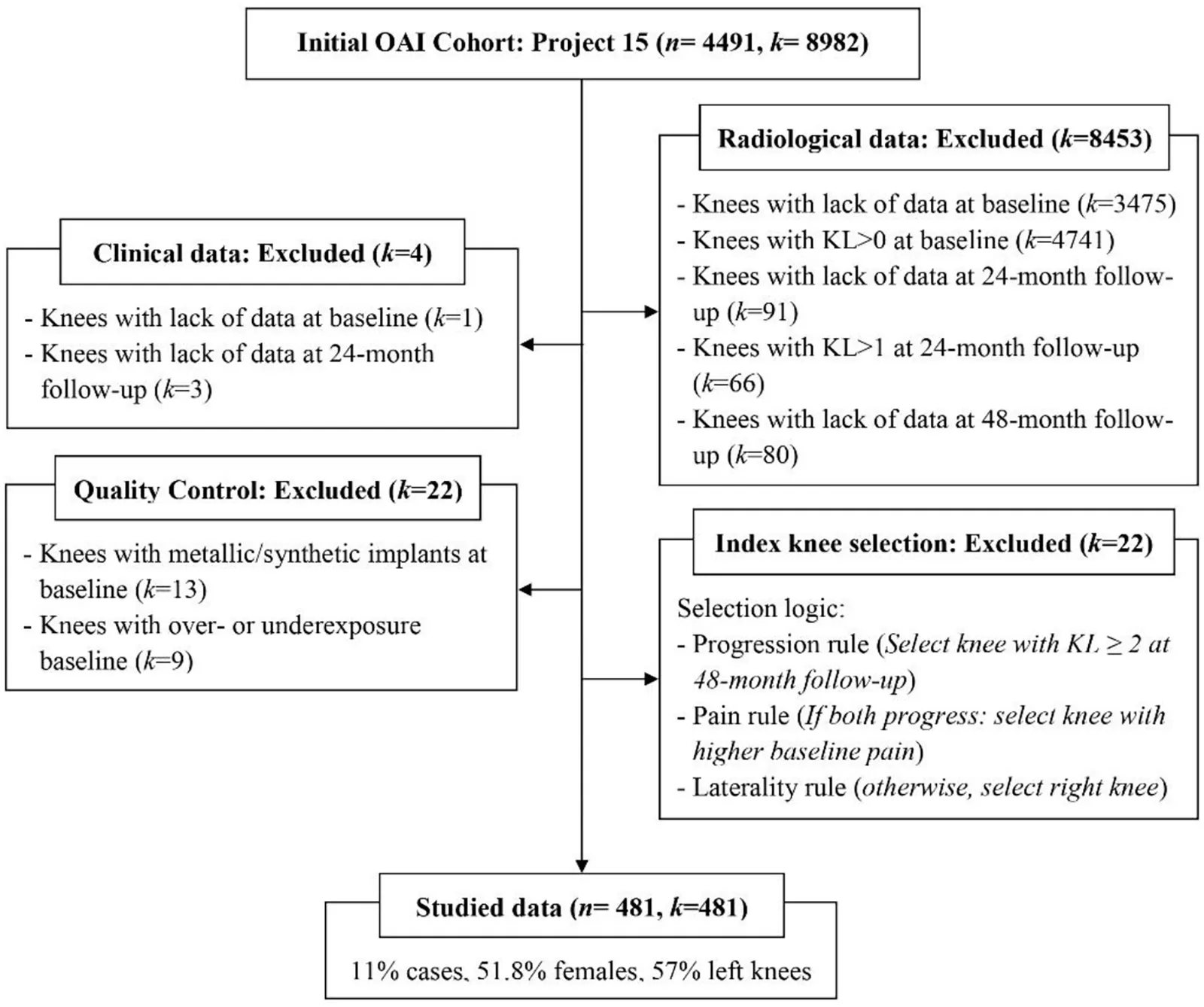
Flow diagram illustrating case–control selection and data inclusion criteria.

#### 2.2.5 Calculation of TBT parameters

Each selected knee radiograph was analyzed to compute the fractal dimension (H) parameters using the quadratic variations estimator (Bousson et al., 2012). As illustrated in **Figure 2**, a total of 16 square-shaped regions of interest (ROIs) were automatically selected over the tibial subchondral bone, covering both medial and lateral compartments (Janvier et al., 2017a). The anatomical landmarks defining the proximal tibial boundaries were identified using the BoneFinder^®^ software (Lindner et al., 2013).

**Figure 2:**
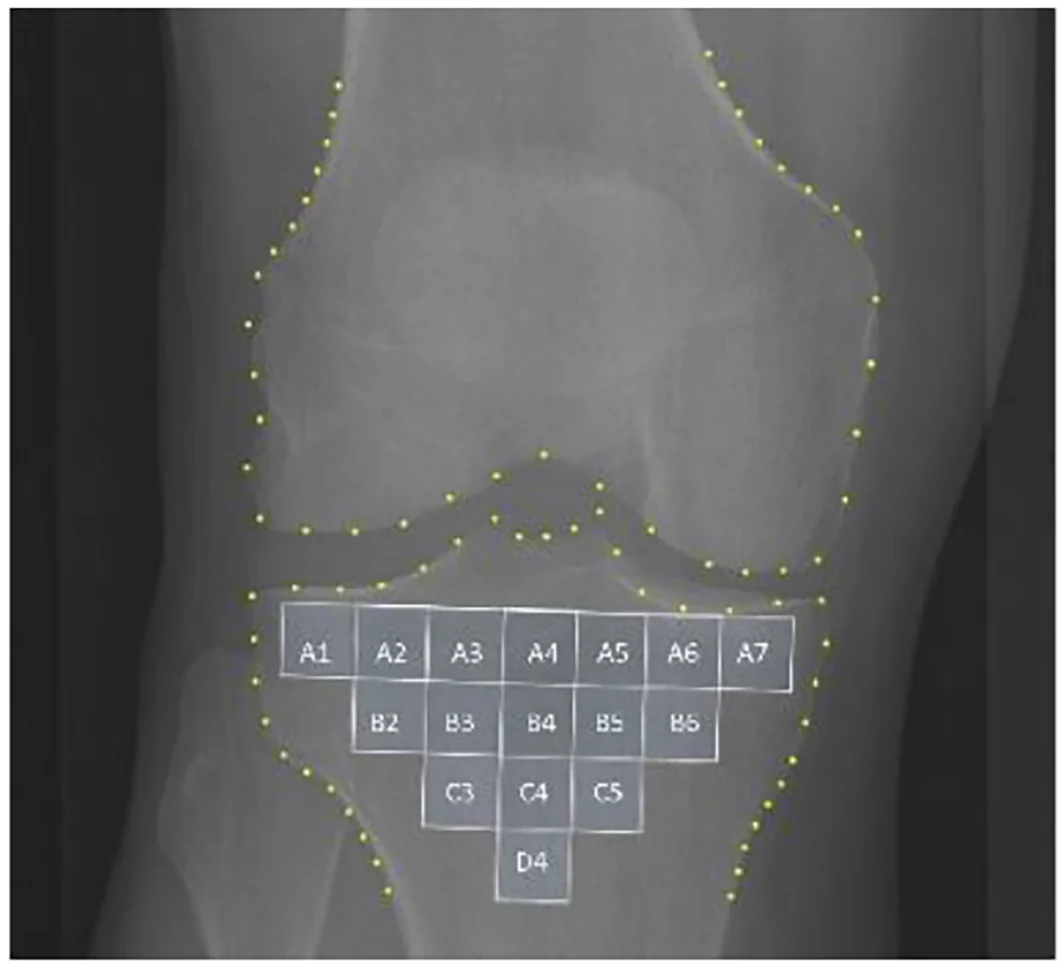
Example of the 16 automatically selected regions of interest covering the entire tibial subchondral bone structure.

For each of the 16 ROIs, TBT was quantified along both vertical and horizontal orientations and across two spatial scales (micro- and milli-scales), yielding 64 texture descriptors per knee. Empirical variograms were used to define the scale separation, with a cut-off identified at approximately 500 μm. Accordingly, two fractal parameters were derived to characterize texture complexity: a micro-scale parameter (μ-scale; <400 μm) and a milli-scale parameter (m-scale; >600 μm), computed along both orientations corresponding to the principal mechanical loading axes of compression (90°) and tension (0°), respectively. No additional image preprocessing, intensity normalization, spatial resampling, or cross-modality harmonization was applied prior to texture analysis. Almhdie-Imjabbar et al. previously evaluated TBT-based model performance in CR and RG images and reported no detectable effect of acquisition modality (Almhdie-Imjabbar et al., 2022a).

#### 2.2.6 Longitudinal variations

Baseline to 24-month variations (Δ) in TBT descriptors as well as in clinical BMI and WOMAC pain readings were calculated using the arithmetic difference between the readings at the 24-months visit and their corresponding readings at baseline.

#### 2.2.7 Statistical analysis

Logistic regression was widely employed to model and predict the incidence of rKOA (Janvier et al., 2017a; Paz-González et al., 2024). As a supervised learning method, it estimates the probability of a binary outcome (case/control) by modeling the log-odds of the dependent variable as a linear combination of predictor variables (Caie et al., 2021). In this study, multivariable logistic regression models including TBT features, adjusted for clinical covariates (CLIN: age, sex, body mass index [BMI], and WOMAC pain score) and radiographic descriptors (KL and medial joint space narrowing [JSNM]), were constructed to evaluate the performance of 24-month longitudinal changes in both TBT (ΔTBT) and clinical parameters (ΔCLIN) for predicting incident rKOA.

In the present study, we evaluated the performance of 5 different prediction models. Model 1 was based on CLIN covariates, and radiological (KL and JSNM) grades, and it was used as a reference model. Models 1 and 2 relied exclusively on baseline data whereas Models 3 to 5 additionally incorporated ΔTBT and/or ΔCLIN descriptors.

- Model 1: CLIN + KL + JSNM (Reference model).
- Model 2: TBT + (Model 1).
- Model 3: ΔTBT + (Model 2).
- Model 4: ΔCLIN + (Model 2).
- Model 5: ΔTBT + ΔCLIN + (Model 2).

In this study, all statistical analyses were performed using the R Statistical tool (version 4.5.1, 2025) and the MASS package (version 7.3-65, 2025). Bidirectional stepwise selection based on the Akaike information criterion (AIC) method (Akaike, 1974), implemented using the MASS package, was applied once to the complete analytical dataset as a dimensionality-reduction procedure for the candidate TBT descriptors, consistent with previous exploratory TBT studies. The selection procedure considered the applicable baseline TBT descriptors and, for models incorporating longitudinal texture changes, the ΔTBT descriptors. Clinical covariates (age, sex, BMI, and WOMAC pain) and radiographic scores (KL and JSNM) were not included in the stepwise-selection procedure; they were subsequently added to the selected TBT model as forced covariates. Feature selection was not repeated independently within the cross-validation training folds. No predictor standardization was applied before model fitting.

For descriptive spatial visualization of the fitted Model 5 coefficients, the intercept and non-estimable coefficients were excluded, and the absolute coefficient of each retained predictor $C_{j}$ was normalized by the sum of the absolute coefficients in that model:


$$C_{j}=|\beta_{j}|/\sum_{k}|\beta_{k}|\times 100$$

The normalized absolute coefficient values corresponding to the selected baseline TBT and ΔTBT descriptors were mapped to their respective regions of interest. A descriptive threshold of *C >*2% was used to highlight coefficient patterns in the spatial maps. Because predictors were not standardized and several descriptors were correlated, *C* was not interpreted as a scale-independent measure of predictor importance, statistical significance, independent contribution, or percentage of model discrimination.

Model discrimination was assessed using the area under the receiver operating characteristic curve (AUC). AUC summarizes the ability of a model to rank incident knees above non-incident knees across possible decision thresholds; values approaching 1 indicate stronger discrimination, whereas a value of 0.5 indicates no discrimination (Bradley, 1997; Mandrekar, 2010; Hosmer et al., 2013). Because AUC does not describe classification performance at a specific operating threshold and may not fully reflect minority-class performance in an imbalanced dataset, it was interpreted together with complementary threshold-dependent metrics. Complementary threshold-dependent metrics included sensitivity, specificity, balanced accuracy (BACC), positive predictive value (PPV), negative predictive value (NPV), and F1 score. BACC was calculated as the mean of sensitivity and specificity, while the F1 score was calculated as the harmonic mean of sensitivity and PPV. These metrics were calculated using the conventional predicted-probability threshold of 0.5. No data-driven threshold optimization was performed. Further details on the definition of these parameters are provided in work (Powers, 2011).

The resulting fixed model specifications were evaluated using stratified 10-fold cross-validation, with class proportions maintained within each fold. Cross-validation was repeated 300 times, and the resampled predictions were summarized to estimate model discrimination and classification performance. Because AIC-based feature selection was performed before rather than within cross-validation, this procedure evaluated the fixed selected models and did not account for uncertainty or potential optimism introduced by feature selection. No constraint based on the number of incident events per estimated parameter was imposed, and penalized-regression methods such as ridge, lasso, or elastic-net regression were not evaluated.

## 3 Results

Following the inclusion/exclusion criteria illustrated in **Figure 1**, a total of 481 knees (57% are left) were considered eligible for the current study. **Table 1** summarizes the clinical characteristics of the study population.

**Table 1:** Characteristics of the datasets included in this study.

Characteristics	Controls	Cases	Total
*@ baseline*			
No. of knees	429	52	481
Age (years)	61.9(±9.1)	60.0(±8.0)	61.7(±9.0)
Females	50.3%	63.5%	51.8%
Males	49.7%	36.5%	48.2%
BMI (kg/m^2^)	28.0(±4.0)	28.6(±5.5)	28.1(±4.2)
*At 24-month follow-up*			
No. of knees	429	52	481
WOMAC pain	1.3(± 2.5)	1.5(± 2.3)	1.3(± 2.5)
BMI (kg/m^2^)	28.1(±4.1)	28.7(±5.5)	28.2(±4.3)
WOMAC pain	1.2(± 2.3)	2.3(± 2.6)	1.3(± 2.3)

Values for Age, BMI and WOMAC pain are represented as mean (± standard deviation).

The present study investigated whether longitudinal variations in trabecular bone texture (ΔTBT) and clinical parameters between baseline and 24 months could improve the prediction of the incidence of rKOA over the subsequent 24 months (24–48 months). TBT features were quantified across micro- and milli-scales defined by a 500 μm cut-off derived from empirical variograms and computed along vertical and horizontal orientations corresponding to the principal loading axes of compression and tension, respectively. According to this definition, **Table 2** summarizes the predictive performances of all models and **Figure 3** reported the corresponding ROC curves. DeLong’s test was used to compare the AUC of each model with that of the reference model, composed of clinical and radiological descriptors only. Models based on baseline TBT descriptors and their 24-monthn longitudinal variations (TBTΔTBT) showed a highly statistically significant increase in AUC (DeLong’s test, pValue < 0.001), demonstrating enhanced discriminative performance relative to the reference model.

**Table 2:** Performance results using trabecular bone texture (TBT) analysis of the whole tibial subchondral zone.

Ref.	Models	BACC	PPV	NPV	TPR	TNR	F1	AUC	*pValue*
Model#1	CLIN+KL+JSNM*	0.50	NE	0.89	0.00	1	NE	0.53 (0.441-0.627)	
Model#2	TBT+Model#1	0.51	0.31	0.89	0.04	0.99	0.07	0.64 (0.557-0.719)	0.0550
Model#3	ΔTBT+Model#2	0.55	0.22	0.90	0.19	0.92	0.20	**0.76** (0.703-0.816)	2.8e-05
Model#4	ΔCLIN+Model#2	0.53	0.23	0.90	0.07	0.98	0.11	0.65 (0.573-0.730)	0.0252
Model#5	ΔTBT+ΔCLIN+Model#2	0.56	0.22	0.90	0.20	0.91	0.21	**0.76** (0.702-0.816)	4.2e-05

Sensitivity (TPR), specificity (TNR), balanced accuracy (BACC), positive predictive value (PPV), negative predictive value (NPV), and F1 score were calculated using a predicted-probability threshold of 0.5. For the reference model, no knees were classified as incident; sensitivity was therefore zero, while PPV and F1 score were not estimable (NE). AUC summarizes ranking discrimination across possible thresholds. The highest AUC value is shown in bold, and the asterisk identifies the reference model.

**Figure 3:**
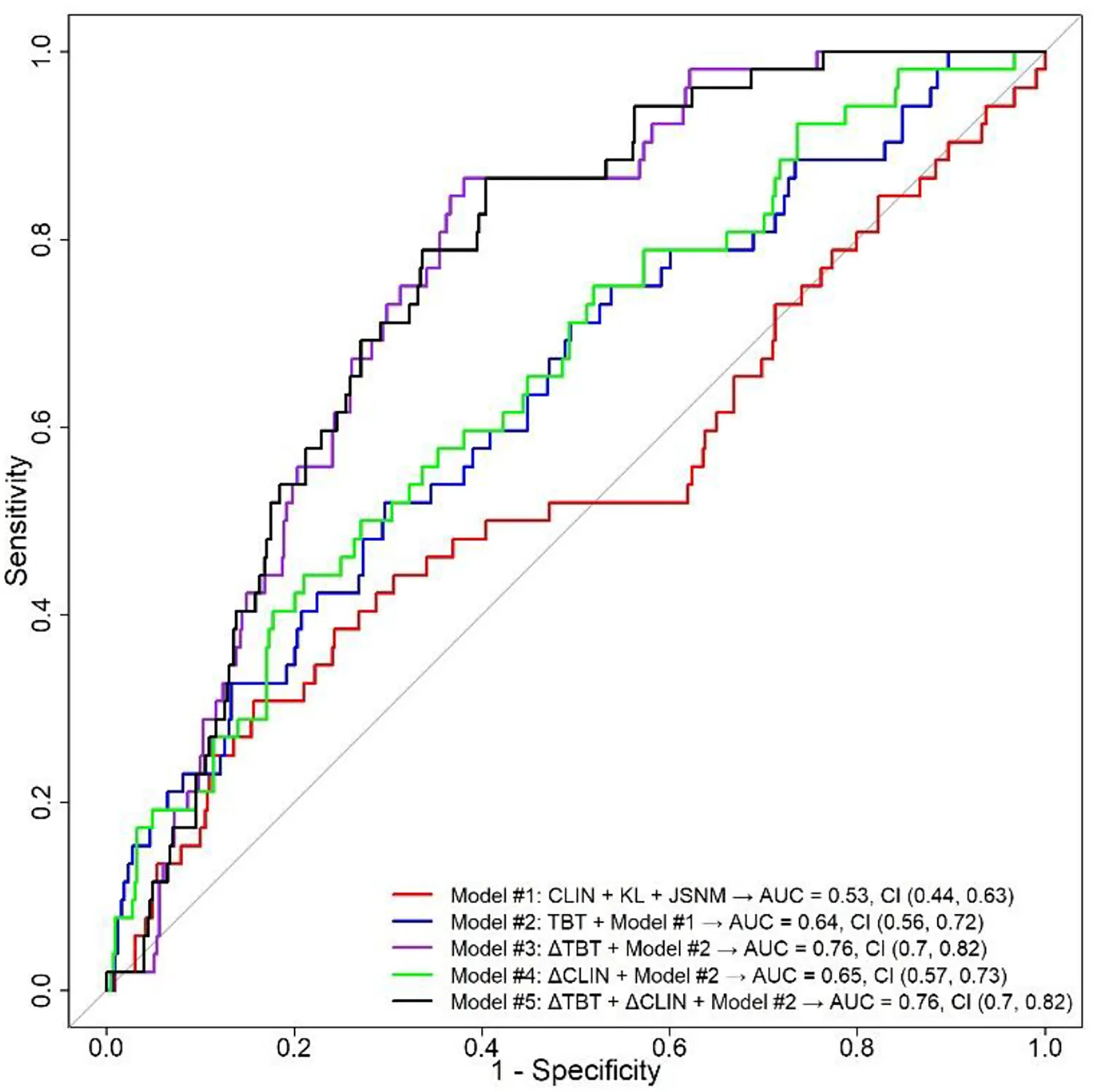
Receiver operating characteristic (ROC) curves of the five studied prediction models evaluated for incident radiographic knee osteoarthritis.

Models 3 and 5 yielded the highest AUC values, indicating higher apparent ranking discrimination than the reference model. However, their classification performance at the 0.5 threshold remained modest. Model 5 had a balanced accuracy of 0.56, PPV of 0.22, and F1 score of 0.21. The reference model did not classify any knee as incident at this threshold; consequently, its sensitivity was zero, while PPV and F1 score were not estimable.

In the present exploratory analysis, models incorporating baseline TBT and ΔTBT yielded an AUC of 0.76 (95% CI 0.70–0.82), compared with 0.53 (95% CI 0.44–0.63) for the clinical and radiographic reference model (p<0.001). This difference concerned ranking discrimination across possible thresholds and was not accompanied by comparably strong classification performance at the 0.5 threshold. Moreover, because TBT selection was performed using the complete dataset before cross-validation, these estimates may be optimistic.

The AIC procedure applied to the complete analytical dataset selected a subset of baseline TBT and ΔTBT descriptors for inclusion in Model 5. The descriptive spatial distributions of their normalized absolute coefficient values (*C*) are presented in **Figures 4**, **5**. Because the predictors were not standardized and several descriptors were correlated, these values were not used to quantify the independent predictive contribution of baseline TBT, ΔTBT, or the clinical and radiographic covariates.

**Figure 4:**
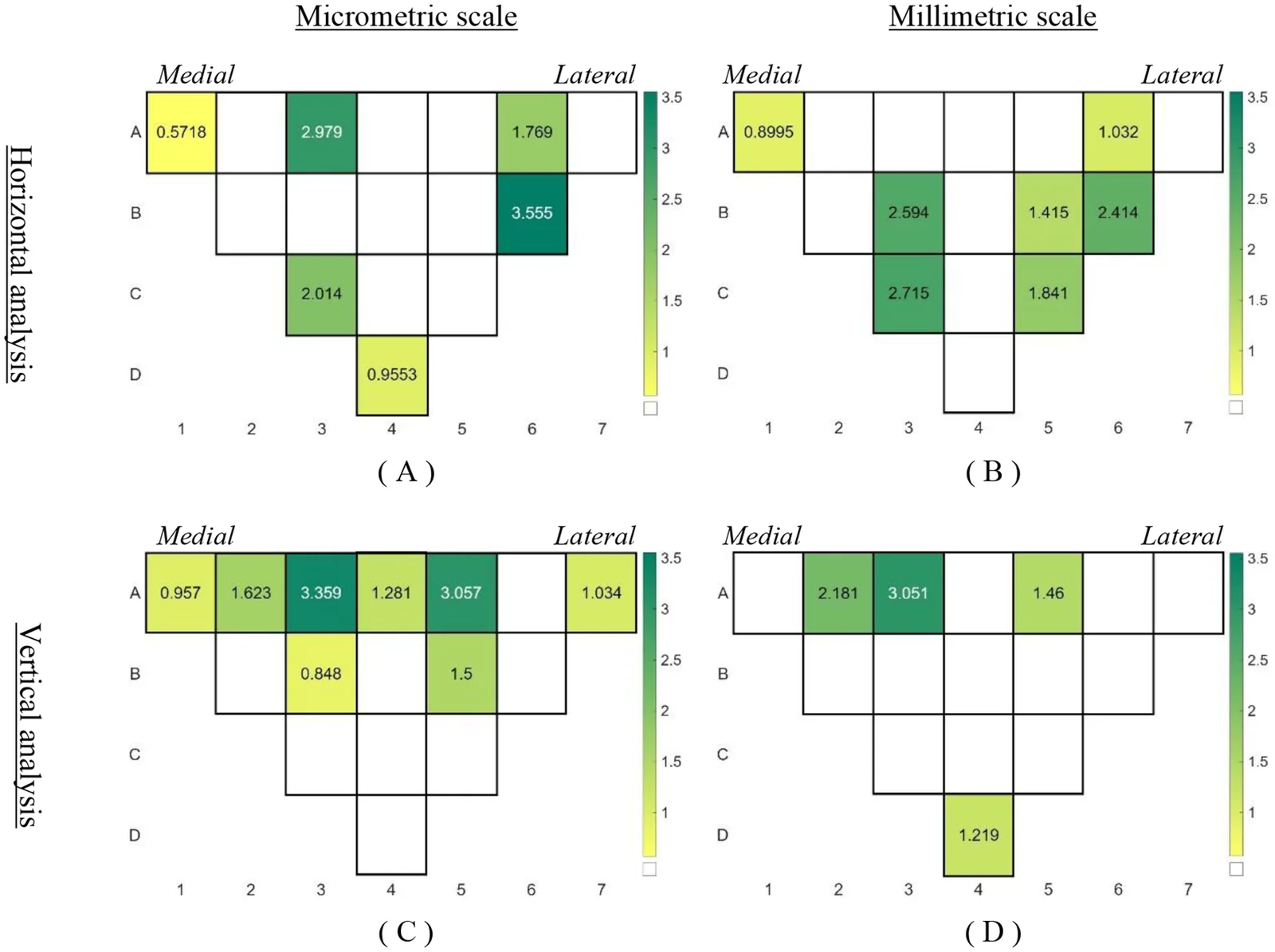
Descriptive spatial distribution of normalized absolute coefficient values for baseline TBT descriptors selected in fitted Model 5: **(A)** horizontal analysis at the micrometric scale; **(B)** horizontal analysis at the millimetric scale; **(C)** vertical analysis at the micrometric scale; and **(D)** vertical analysis at the millimetric scale. The maps describe the fitted full-dataset model and do not represent held-out or independent predictor importance.

**Figure 5:**
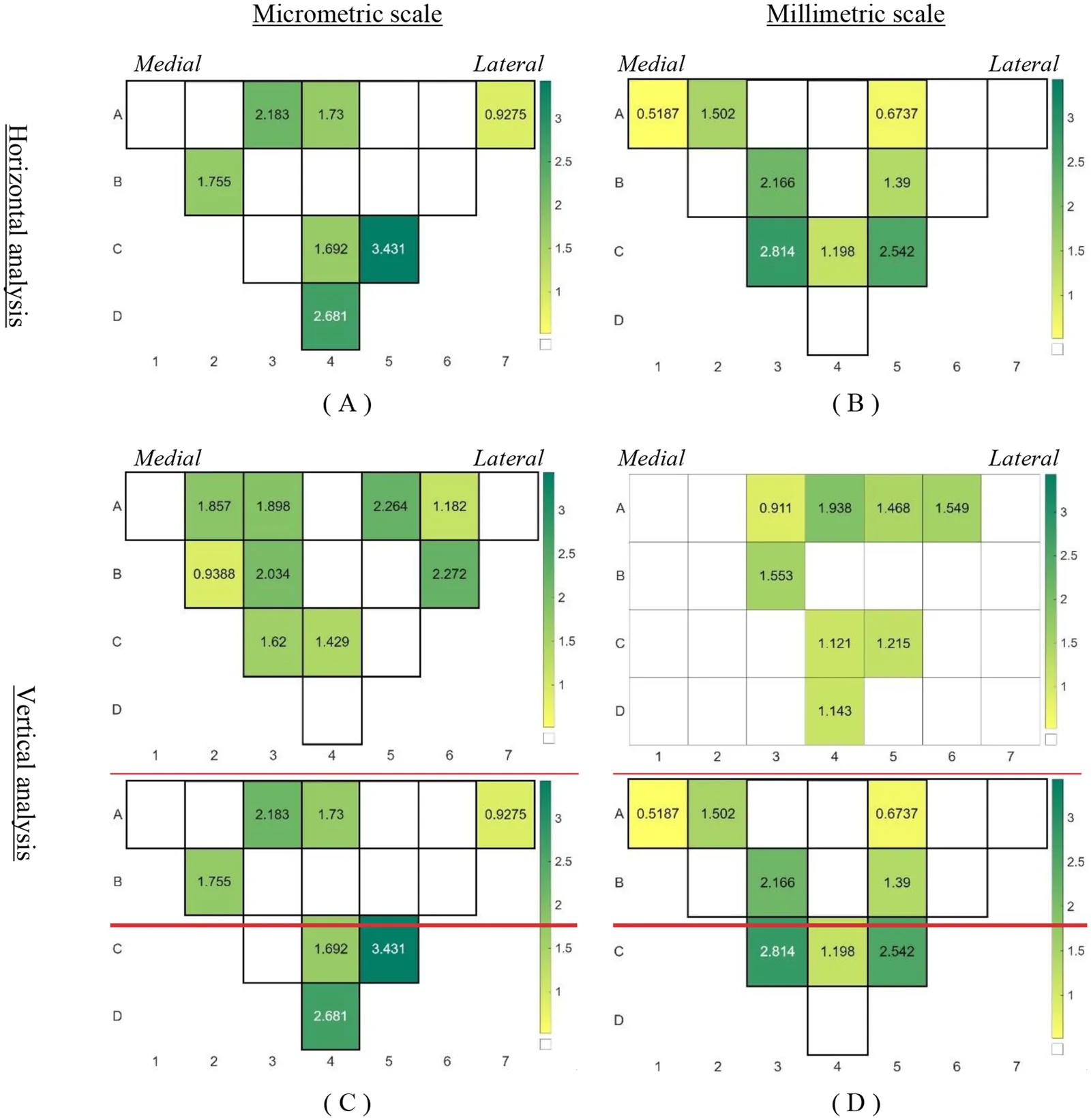
Descriptive spatial distribution of normalized absolute coefficient values for longitudinal ΔTBT descriptors selected in fitted Model 5: **(A)** horizontal analysis at the micrometric scale; **(B)** horizontal analysis at the millimetric scale; **(C)** vertical analysis at the micrometric scale; and **(D)** vertical analysis at the millimetric scale. The maps describe the fitted full-dataset model and do not represent held-out or independent predictor importance.

**Figure 4** presents the descriptive spatial distribution of normalized absolute coefficient values (*C*) for the baseline TBT descriptors selected in fitted Model 5. Descriptors with *C*>2% were located primarily in the medial compartment, including ROI A3 across several analytical scales and orientations. Selected descriptors with *C*>2% were also observed in the lateral compartment, including ROIs A5 and B6. These locations describe the coefficient pattern of the fitted full-dataset model and should not be interpreted as stable or independently validated predictor importance.

**Figure 5** presents the descriptive spatial distribution of normalized absolute coefficient values (*C*) for the ΔTBT descriptors selected in fitted Model 5. For micro-scale horizontal descriptors, values above the descriptive threshold of *C*>2% were observed in ROIs A3, C5, and D4. For micro-scale vertical descriptors, values above this threshold were observed in ROIs A5, B6, and B3. For milli-scale horizontal descriptors, ROIs B3, C3, and C5 exceeded the descriptive threshold. No milli-scale vertical ΔTBT descriptor had a *C* value above 2%. These spatial coefficient patterns are descriptive and hypothesis-generating and do not establish the independent predictive or biological importance of the corresponding regions.

These spatial coefficient patterns are descriptive and hypothesis-generating and should not be interpreted as stable or independently validated predictor importance.

## 4 Discussion

In this exploratory analysis, a large set of radiographic texture descriptors was extracted from baseline TBT measurements and their changes over 24 months. Models incorporating these descriptors yielded an AUC of 0.76 for incident rKOA in the present OAI-derived sample. However, because feature selection preceded cross-validation, this estimate may overstate the performance expected in new data. Descriptive coefficient mapping showed that selected baseline and longitudinal TBT descriptors were distributed across anatomically defined regions of the tibial subchondral bone. However, these coefficient values cannot establish that TBT provided more independent predictive information than the clinical or radiographic variables. The observed spatial distribution of selected ΔTBT descriptors generates hypotheses regarding dynamic subchondral bone remodeling in early rKOA. However, the instability risk associated with the high-dimensional stepwise procedure prevents definitive attribution of predictive or biological importance to individual descriptors.

Several selected descriptors with larger normalized absolute coefficients were located in the medial subchondral tibial plateau, particularly ROI A3, implicating both superficial and deep trabecular layers in early OA processes. A single lateral ROI (B6) also contributed, supporting the notion that lateral-compartment OA, while less common, may arise as a distinct phenotype (Zhang et al., 2025). Milli-scale vertical descriptors generally had smaller normalized absolute coefficients in the fitted model. However, coefficient magnitude alone is insufficient to conclude that these trabecular patterns lacked predictive or biological relevance.

In the present exploratory analysis, models incorporating ΔTBT yielded higher apparent ranking discrimination than the clinical and radiographic reference model, with an AUC of approximately 0.76. However, classification performance at the 0.5 threshold remained modest: the best-performing model had a balanced accuracy of 0.56, PPV of 0.22, and F1 score of 0.21. The p-value below 0.001 concerned the AUC comparison and should not be interpreted as evidence of strong classification performance or clinical usefulness. Short-term clinical changes did not produce an observable improvement in the evaluated models. These findings suggest that longitudinal subchondral texture changes may contain information relevant to incident rKOA, but this interpretation requires validation using a leakage-free modeling pipeline and an independent cohort.

A previous work by Janvier et al. reported an AUC of 0.69 for predicting incident radiographic knee osteoarthritis using a combination of clinical variables and TBT descriptors derived from 344 conventional radiographs from 319 participants (Janvier et al., 2017a). While the present study extends this framework, they are different in several methodological issues. First, we focused on spatially resolved longitudinal changes in subchondral TBT, thereby capturing dynamic microarchitectural remodeling rather than static baseline patterns alone. Second, we enforced a strict one-knee-per-participant inclusion criterion, thereby avoiding within-participant correlation between two knees. However, the hierarchical rule used to select between two eligible knees relied on 48-month progression status, which may have introduced outcome-dependent selection bias. Third, we included both computed (CR) and digitized (RG) radiography acquisitions, whereas the study by Janvier et al. included computed radiography only (Janvier et al., 2017a). Radiographic texture measurements may be influenced by image quality and acquisition characteristics. In the OAI, participants were followed at the same clinical center, and serial radiographs were acquired using a standardized fixed-flexion protocol with consistent acquisition modality and image resolution across visits. Because ΔTBT was calculated within each knee, this longitudinal consistency reduced the influence of time-invariant center-, modality-, and resolution-related characteristics. Moreover, Almhdie-Imjabbar et al. specifically evaluated the influence of CR and RG images on TBT-based model performance and found no detectable effect of acquisition modality (Almhdie-Imjabbar et al., 2022a). In addition, Acquisition modality and pixel spacing remained consistent between baseline and 24 months for all included knees, reducing the potential influence of modality changes on ΔTBT. Nevertheless, residual variability related to exposure, positioning accuracy, equipment calibration, grayscale response, and image quality cannot be completely excluded. External validation across independent cohorts and imaging systems is therefore required (Janvier et al., 2017a; Almhdie-Imjabbar et al., 2023a).

This study has several strengths. Longitudinal radiographs combined with high-resolution TBT analysis allowed detection of early microarchitectural changes, providing explanatory insight by linking predictive features to anatomically relevant subchondral regions. Selecting a single knee per participant avoided correlation between observations from the same participant. However, because the index-knee rule incorporated 48-month progression status, the resulting predictions should not be interpreted as unbiased person-level risk estimates (El-Zaatari et al., 2025). Our definition of incident rKOA was aligned with recent reviews (Almhdie-Imjabbar et al., 2023a) and adapted to capture early structural changes most relevant for rKOA prediction. The coefficient-based spatial maps provided a descriptive visualization of the fitted model, although the stability and independent predictive contribution of the selected descriptors were not established.

The limitations of this study include restricting analysis to one knee per participant, which may limit statistical power relative to paired-knee studies. An important methodological limitation is that AIC-based TBT selection was performed once using the complete analytical dataset before cross-validation. Consequently, outcome information from observations subsequently assigned to held-out folds contributed to selection of the TBT descriptors. Index-knee selection may represent a source of bias because, when both knees were eligible, the knee progressing to KL grade ≥2 was preferentially selected. Although including one knee per participant avoided within-participant correlation, the resulting case proportion and predictive estimates should be interpreted within the selected analytical sample. Future studies could use an outcome-independent selection rule or include both knees using participant-level statistical methods. Predictors were not standardized before model fitting. Although this does not alter the fitted probabilities of an unpenalized logistic-regression model under ideal numerical conditions, it limits direct comparison of coefficient magnitudes across variables measured on different scales. The cross-validation procedure therefore evaluated fixed, previously selected model specifications rather than the complete feature-selection and model-fitting pipeline, potentially producing optimistic performance estimates. This concern is particularly relevant given the 52 incident cases and the large number of candidate baseline and longitudinal TBT descriptors. Repeating feature selection independently within each training fold would provide stronger protection against information leakage and would allow feature-selection stability to be quantified. The present results should therefore be considered exploratory and require validation in an independent cohort using a prespecified, locked feature set. The discrepancy between AUC and threshold-dependent metrics indicates that the models showed moderate apparent ranking discrimination but limited positive classification performance at the conventional 0.5 threshold. The reference model did not classify any knee as incident, while the TBT-containing models had low PPV and F1 scores. No clinically meaningful operating threshold was prespecified, and no data-driven threshold optimization, calibration analysis, or decision-curve analysis was performed. Consequently, these results do not support individual-level clinical decision-making. External validation should include assessment of calibration, precision–recall performance, and net clinical benefit at clinically justified operating thresholds. Radiographic TBT measurements are also sensitive to image quality and acquisition characteristics, including scanner type, spatial resolution, pixel size, exposure, digitization, grayscale response, positioning, and image post-processing. The study included both computed radiography and digitized conventional radiography without additional image harmonization or explicit adjustment for these technical factors. Although OAI radiographs were acquired under standardized protocols and centralized quality control, residual technical variability may have influenced both baseline TBT and ΔTBT measurements. The quadratic-variations estimator has previously demonstrated robustness in OAI radiographs and has been applied in an independent study (Almhdie-Imjabbar et al., 2022a); however, this evidence does not establish complete invariance across acquisition modalities or imaging systems. Within-knee longitudinal differences may attenuate participant-specific and time-invariant acquisition effects when imaging conditions remain consistent across visits, but they cannot eliminate variability arising from changes in acquisition or digitization conditions. Future studies should assess modality consistency between visits, account for relevant technical imaging characteristics, and externally validate TBT measurements across independent imaging systems. Systemic factors, such as metabolic or inflammatory status, which can influence bone remodeling, were not incorporated. The absence of MRI-based biomarkers represents another limitation, as they could provide complementary information and enhance early detection (Roemer et al., 2022). Expanding the dataset to include external cohorts, such as MOST, would improve sample size and generalizability. Furthermore, the predominance of medial-compartment OA in this study limits conclusions regarding lateral-compartment progression. While a lateral ROI contributed to prediction, the study was not specifically designed to examine lateral OA development. Finally, the reported discrimination was moderate, while threshold-dependent performance was limited. Given the non-nested feature-selection procedure and absence of external validation, the reported performance should not be interpreted as evidence of clinical utility.

The limited number of incident knees relative to the number of candidate descriptors represents an additional limitation. Our of the 128 TBT and ΔTBT descriptors, up to 57 were considered with only 52 incident knees, creating a risk of unstable selection, imprecise coefficients, and overfitting. AIC-based reduction does not eliminate these concerns, and selection stability was not evaluated. Future studies should assess penalized models within nested resampling and report feature-selection frequencies across training samples.

Collectively, these exploratory findings support further investigation of spatially resolved ΔTBT as a candidate radiographic imaging marker of incident rKOA. The results generate hypotheses regarding the role of dynamic subchondral bone remodeling in early disease development but do not establish individual-level clinical utility. Leakage-free internal validation and external validation in independent cohorts [e.g., MOST (Segal et al., 2013) and KHOALA (Guillemin et al., 2012)] will be required before ΔTBT can be considered for risk stratification, clinical monitoring, or trial enrichment.

## 5 Conclusions

In this exploratory OAI analysis, the selected baseline and longitudinal TBT descriptors were spatially concentrated primarily in the medial subchondral tibial compartment across superficial and deep layers. This spatial distribution provides hypotheses for future investigation but should not be interpreted as a stable predictive signature. Models incorporating ΔTBT showed higher apparent discrimination than the clinical and radiographic reference model. However, because TBT selection was performed using the complete dataset before cross-validation, the magnitude of this improvement may be optimistic, and threshold-dependent classification performance remained limited. These findings support further investigation of spatially resolved ΔTBT as a candidate imaging marker but do not establish clinical utility. Validation using penalized modeling, leakage-free resampling, and independent cohorts and imaging systems is required to establish the stability, reproducibility, and generalizability of longitudinal TBT measurements.

## Data Availability

The data supporting the findings of this study are available from the Osteoarthritis Initiative (https://nda.nih.gov/oai). Access to these data is subject to restrictions, as they were obtained under license for the present study and are therefore not publicly accessible in full. However, the data used in this analysis can be made available by the authors upon reasonable request and with permission from the Osteoarthritis Initiative.
